# DAILY STRESS AND SAME-DAY SUBJECTIVE MEMORY: AGE DIFFERENCES AND PHYSICAL ACTIVITY AS A BUFFER

**DOI:** 10.1093/geroni/igad104.1305

**Published:** 2023-12-21

**Authors:** Nicole Stuart, Jin Wen, Patrick Klaiber, Anita DeLongis, Eli Puterman, Nancy Sin

**Affiliations:** University of British Columbia, Vancouver, British Columbia, Canada; University of British Columbia, Vancouver, British Columbia, Canada; Tilburg University, Tilburg, Noord-Brabant, Netherlands; University of British Columbia, Vancouver, British Columbia, Canada; University of British Columbia, Vancouver, British Columbia, Canada; University of British Columbia, Vancouver, British Columbia, Canada

## Abstract

Growing research indicates that daily stress is associated with poor same-day memory performance, but it is unclear whether this relationship varies by age and whether moderate-to-vigorous physical activity (MPVA) might offset the within-person link between stress and memory in daily life. Ecological momentary assessment data were collected from adults aged 25-88 across British Columbia, Canada. For 14 days, participants (N = 249) wore a tri-axial physical activity monitor, reported stressor occurrence and perceived stressfulness of the events in mobile surveys 4 times per day, and rated their memory at the end of each day. Multilevel modeling was run to evaluate daily perceived stressfulness as a predictor of subjective memory, with same-day MVPA engagement (activity counts >570 counts/60s) and age (years) entered as moderators. At the within-person level, on days when stressors were perceived as more stressful than usual, subjective memory was rated as relatively worse (b = -0.037, CI[-0.061, -0.017], p < .001). MVPA did not buffer against the within-person associations of daily stress with subjective memory, nor did age (p > .05). Future work could examine the mechanisms that might explain the link between stress and memory, for example cortisol, as well as the associations of different intensity and forms of physical activity on stress across age groups.

